# Neurophysiological Assessment of F-Wave, M-Wave, and Cutaneous Silent Period in Patients with Caput-Pattern Cervical Dystonia at Waning and Peak Response Phases of Botulinum Toxin Therapy

**DOI:** 10.3390/toxins18010021

**Published:** 2025-12-30

**Authors:** Artur Drużdż, Edyta Leśniewska-Furs, Małgorzata Dudzic, Anna Sowińska, Szymon Jurga, Wolfgang H. Jost

**Affiliations:** 1 Department of Neurology, Municipal Hospital, 61-285 Poznań, Poland; edyta_lesniewska@wp.pl (E.L.-F.); gosiadudzic@gmail.com (M.D.); 2 Department of Computer Science and Statistics, Poznan University of Medical Sciences, 60-806 Poznań, Poland; ania@ump.edu.pl; 3 Department of Neurology, Collegium Medicum, University of Zielona Góra, 65-046 Zielona Góra, Poland; szymon.jurga@vp.pl; 4 Parkinson-Klinik Ortenau, 77709 Wolfach, Germany; w.jost@parkinson-klinik.de

**Keywords:** cervical dystonia, f-wave, cutaneous silent period, botulinum toxin, Col-Cap concept

## Abstract

While distinguishing between collis and caput patterns in cervical dystonia (CD) has clear clinical and therapeutic relevance, the effects of botulinum toxin type A (BoNT-A) on segmental spinal excitability and inhibitory function in caput-pattern CD have not been previously investigated. This study aimed to advance understanding of the effects of BoNT-A and its broader neurophysiological impact in cervical dystonia, particularly in the caput subtype. The study utilised non-invasive neurophysiological methods to assess F-wave and cutaneous silent period (CSP or CuSP) parameters in 21 CD patients with caput motor patterns at waning and peak response phases of BoNT-A therapy. Significant prolongation of Fmin latency, increased F–M interlatency, reduced F-wave amplitude, and a marked increase in CSP duration and onset latencies were observed following BoNT-A administration, indicating that BoNT-A not only reduces spinal motoneuron excitability and strengthens spinal inhibitory processes, but also highlights its capacity to modulate central sensorimotor pathways beyond local chemodenervation. Together, the observed changes in CSP support its use as a potential biomarker for nervous system effects of BoNT-A in dystonia; however, further validation in controlled studies is warranted.

## 1. Introduction

Cervical dystonia (CD) is a condition characterised by involuntary, sustained or intermittent contractions of cervical muscles, resulting in abnormal movements or postures of the head and neck [1]. The Col-Cap concept is based on distinguishing between muscles that act primarily on the head (caput) and those that act on the neck (collis). This distinction has proven particularly valuable in guiding botulinum toxin type A (BoNT-A) injections into the involved muscles, which is the first-line treatment for CD. The caput patterns involve dystonic muscles located above the C3 vertebra (rostral to C3), causing movements of the head relative to the neck: pivotal movement called torticaput (TCap), lateral flexion called laterocaput (LCap), forward flexion called anterocaput (ACap), and backward extension called retrocaput (RCap). The collis patterns involve muscles below the C2 vertebra (caudal to C2), causing movements in the neck relative to the trunk [2]. Even though the aetiology of cervical dystonia is not yet clear, increasing evidence points to a disorder of sensorimotor integration and impaired inhibitory control within the central nervous system [3]. Neurophysiological studies provide objective tools to investigate these abnormalities.

Two well-established, non-invasive tools that provide insights into various aspects of neural excitability are the F-wave and the cutaneous silent period (CSP). The F-wave, which is a late response elicited by supramaximal electrical stimulation of a motor nerve, reflects the excitability of the anterior horn cells and motor conduction in the proximal and distal segments of the median nerve, the plexus and motor radix C8–Th1 [4,5]. Alterations in F-wave parameters, such as persistence, latency, and chronodispersion, have been observed in movement disorders, including dystonia, and may indicate abnormal spinal motoneuron excitability [6]. In turn, the cutaneous silent period (CSP)—a transient suppression of voluntary muscle activity following a cutaneous noxious stimulus—is considered a spinal and supraspinal inhibitory phenomenon mediated in part by A-delta fibres [7]. Prolongation or attenuation of CSP duration has been reported in dystonia and is consistent with the concept of impaired inhibitory control [8]. Together, these measures provide complementary perspectives on the excitatory and inhibitory dynamics of the central nervous system in CD.

Because abnormal muscle activity is the hallmark of CD, BoNT-A has become the cornerstone of symptomatic management. BoNT-A blocks neuromuscular transmission by inhibiting the release of acetylcholine at cholinergic synapses, which reduces excessive muscle contractions in a targeted, reversible manner, alleviating both abnormal postures and pain and significantly improving patient quality of life [9]. Recommendations first articulated by the American Academy of Neurology and subsequently reflected in European and Polish guidelines now regard BoNT-A as the gold standard for CD treatment [10,11].

Despite the proven clinical and therapeutic relevance of the collis and caput distinction in CD and the theory of sensorimotor integration disorder in this condition [12], to date, no neurophysiological studies have specifically examined the effects of BoNT-A on segmental spinal excitability and inhibitory function across the BoNT-A treatment cycle in patients with caput patterns. What remains underexplored is how these measures vary dynamically across the BoNT-A treatment cycle (i.e., between waning and peak response phases) in patients with caput-pattern CD. Addressing this gap offers an opportunity to expand the understanding of BoNT-A’s effects, particularly on the peripheral and central nervous systems, and may provide further insight into its therapeutic potential and dynamic changes within a BoNT-A treatment cycle, contributing to improved treatment monitoring and optimisation.

This study takes advantage of non-invasive assessment methods to evaluate and compare changes in F-wave and cutaneous silent period (CSP) parameters in patients with cervical dystonia exhibiting caput motor patterns, at waning and peak response phases of BoNT-A treatment, with the aim of detecting and characterising alterations in segmental spinal excitability and inhibitory function associated with this specific dystonia subtype.

## 2. Results

### 2.1. F-Wave and M-Wave Measurements

As shown in Table 1, across most parameters, the differences between the waning and peak phases of botulinum toxin treatment were small. Minimum F-wave latency (Fmin) was slightly higher at the peak phase (increase from 23.70 ± 1.90 ms to 24.25 ± 1.88 ms), and this comparison reached statistical significance before multiple-comparison correction. F-wave persistence and M-wave area showed a similar pattern: both were modestly lower at the peak phase, with uncorrected *p*-values below 0.05. However, none of these findings remained significant after applying the Holm–Bonferroni correction. The remaining F-wave parameters (Fmax, Fmean, chronodispersion and F-wave duration) were comparable between phases, with *p*-values suggesting no meaningful difference. M-wave latency and amplitude also showed no significant change. The effect sizes were generally small, in line with the minimal shifts in mean values. Although a few calculated Cohen’s d values appear large in the table, the underlying descriptive statistics point to only modest differences.

### 2.2. Calculated F-Wave Parameters

As shown in Table 2, the F/M amplitude ratio showed a small decrease at the peak phase, but the difference did not reach statistical significance, and the effect size was moderate. The F/M latency ratio remained almost identical between phases, with no indication of a meaningful change. The latency difference between the F-wave and the M-wave (F–Mlat) was slightly larger at the peak phase (increase from 20.00 ± 1.90 ms to 20.59 ± 1.93 ms). This comparison yielded a statistically significant *p*-value (*p* = 0.022) before correction, but the effect did not hold after Holm–Bonferroni adjustment. The effect size reported in the table is large, although the actual mean difference is small and should be interpreted carefully. Both conduction velocity measures (CV1 and CV2) were modestly lower at the peak phase, but these differences were not statistically significant. Their effect sizes are shown as large, although the underlying mean differences suggest these changes are not significant.

### 2.3. Cutaneous Silent Period (CSP) Measurements

CSP analysis (Table 3) revealed consistent and substantial differences in the duration-related CSP parameters. CSPe and CSPd were both markedly longer at the peak phase (increase from 116.32 ± 10.54 ms to 126.84 ± 7.80 ms and from 38.64 ± 5.89 ms to 48.69 ± 7.33 ms, respectively). These comparisons were highly significant, with *p*-values below 0.0001, and they remained significant after the Holm–Bonferroni correction. The absolute differences in means (around 10 ms for CSPe and about 10 ms for CSPd) support a clear separation between phases. CSPom also increased at the peak phase (increase from 65.40 ± 12.93 ms to 68.16 ± 11.27 ms). This effect was smaller than in CSPe and CSPd but still reached statistical significance (*p* = 0.009), and it also held after the correction for multiple testing. Although the table lists very large Cohen’s d values, the underlying descriptive data simply indicate moderate-to-large differences that are consistent across duration-based CSP measures. In contrast, CSP onset latency (CSPo) was similar in both phases of the treatment. The means and medians differed only slightly, and the statistical comparison did not show any significant change.

### 2.4. CSP Conduction Velocities

As shown in Table 4, sensory conduction velocity (CV3) did not differ meaningfully between phases (*p* = 0.244). Both the mean values and the interquartile ranges were nearly identical, and the comparison was not statistically significant. In contrast, the remaining parameters showed clear and consistent differences. Motor conduction velocity (CV4) was markedly lower at the peak phase, with an absolute drop of more than 4 m/s (21.88 ± 4.00 m/s to 17.26 ± 2.57 m/s,). This difference was highly significant (*p* < 0.0001) and remained so after Holm–Bonferroni correction. Total conduction velocity (CV5) showed a similar pattern: values were clearly lower at the peak phase, with strong statistical support (from 1.486 ± 0.199 m/s to 1.355 ± 0.141 m/s, *p* < 0.0001). The effect sizes listed as Cohen’s d are very large in the table, but they mainly indicate that these differences were consistent and sizeable in this sample.

## 3. Discussion

This study aimed to evaluate the impact of BoNT-A on segmental spinal cord circuits in patients with CD exhibiting the caput pattern. The main findings demonstrate that BoNT-A administration led to significant neurophysiological changes in spinal excitability and sensorimotor inhibition, as reflected by alterations in CSP parameters.

The prolongation of Fmin latency, increase in F–M interlatency (difference between F-wave and M-wave latency), and reduction in F-wave amplitude observed at the peak response phase of BoNT-A treatment may collectively indicate decreased excitability of spinal motoneurons. These findings align with previous reports indicating that BoNT-A not only exerts local chemodenervation but may also induce remote changes at the level of the spinal cord via retrograde afferent pathways [3,13]. Chroni et al. [14] similarly observed a reduction in F-wave parameters post-treatment, suggesting altered recurrent excitability of anterior horn cells. In our study, the increase in F–M latency provides further evidence to this and may imply a partial central adaptation following BoNT-A treatment.

One of the most striking effects of BoNT-A in this study was the significant increase in CSP duration (CSPd) resulting from the prolongation of CSP end latency (CSPe) at the peak response phase of BoNT-A treatment. These changes may suggest an enhancement of spinal inhibitory mechanisms following treatment. The CSP is a polysynaptic spinal reflex modulated by thin A-delta afferents and descending supraspinal motor control [15]. An increase in CSP duration at the peak response phase of BoNT-A treatment has been observed in other focal dystonias and may reflect rebalancing of inhibitory circuits disrupted by the dystonic state [8]. Since CSP probably reflects cortical and subcortical inhibitory circuits, our results may suggest that botulinum toxin may indirectly modulate inhibitory processes.

Interestingly, the results show significant alterations in CSP-related conduction velocities, including a reduction in motor (CV4) and total (CV5) velocities. These changes may indicate altered processing of efferent input and suggest a slowdown of signal propagation following treatment. While counterintuitive at first, this pattern may represent improved gating or delay caused by reactivated inhibitory interneurons within the spinal cord [16]. Sensory conduction velocity CV3 did not decrease significantly (*p* = 0.244) in A-delta fibres. Because these differences remain significant after multiple-comparison correction and show consistent shifts in several metres per second, the results represent a robust physiological change rather than random fluctuation. These integrated measures may offer enhanced insight into the dynamic balance between excitation and inhibition in CD. Their responsiveness to BoNT-A supports their potential utility as biomarkers for treatment monitoring in clinical and research settings.

In this study, BoNT-A injections were administered in accordance with the Col-Cap classification, which distinguishes dystonic activity in the cervical (Col) and cranio-cervical (Cap) muscle groups [17]. The precise, anatomy-driven approach to both patient classification and BoNT-A administration may be reflected in the highly consistent neurophysiological changes observed across the patient group. Given the predominance of caput-type dystonia in the cohort, the results may particularly reflect changes in bulbospinal and upper cervical segments, because the muscles responsible for the caput patterns are innervated by the cranial nerve XI (accessory nerve), nerves originating at the upper cervical spinal segment levels C1–C5 and, in the case of semispinalis capitis, at C2–Th2. Stimulation was applied to the median nerve, emerging from the C6–Th1 levels [18], which is below the level of the innervation of muscles that were injected with BoNT-A. Therefore, in the CSP measurements (where no statistical significance was found for A-delta afferent fibres), the prolonged efferent processing times likely originated from regions superior to the C5 spinal segment, involving the upper cervical segments of the spinal cord, medulla oblongata, and associated sensory-motor tracts. The activation of alpha motoneuron responses subsequently followed this. Therefore, it may be concluded that, following intramuscular administration, BoNT-A is transported along neural fibres to the spinal cord, where it modulates neural activity both at the level of the nerve or nerve root, innervating the targeted muscle or by influencing long and short spinal pathways. The alterations in muscle behaviour induced by the botulinum toxin injection subsequently impact the activity of the spinal segment connected to the treated muscle. As a result, there is a reduction in neural activity not only within the corresponding segment of the spinal cord but also along ascending and descending tracts, reflecting a broad modulatory effect on spinal and supraspinal pathways. Cortical studies in dystonia further support this broad impact of BoNT on central neural circuits. For instance, intramuscular BoNT injections normalised enhanced somatosensory-evoked potentials in patients with cervical dystonia [19] and helped to restore both sensory and motor cortical excitability, as well as intracortical inhibition [20]. The present findings reinforce the view that BoNT-A, beyond acting locally, can induce important changes in how the spinal cord controls muscles at different levels, leading to greater inhibition in several muscle groups. This general increase in inhibition may be functionally significant and may help to restore a normal balance between muscle activation and inhibition in patients with dystonia, improving both local and distant muscle control.

Based on the preliminary findings of the neurophysiological assessments and the concepts cited in the literature, it may be inferred that the prolongation of neural conduction time observed at the peak effect of BoNT-A in CD is more likely to reflect central rather than peripheral mechanisms. This interpretation is supported by normal sensory conduction parameters and only very limited statistical significance in selected F-wave measures, i.e., parameters primarily related to peripheral afferent and efferent fibres. Further support comes from the highly statistically significant prolongation of CSP duration (CSPd) and its end component (CSPe). However, this inference is based on indirect measures, as the CSP parameter cannot be reliably divided into distinct central and peripheral components.

Despite the promising findings, this study is limited by its small sample size, single-centre design, and lack of a healthy control group. Furthermore, although both OnabotulinumtoxinA and AbobotulinumtoxinA were used, comparative analysis between them was not feasible due to the limited subgroup size. Future studies should aim to examine larger, multicentre cohorts that are stratified by toxin formulation and include matched controls to validate and expand these results.

## 4. Materials and Methods

This prospective study was conducted at the Neurology Department of the Municipal Hospital in Poznań (17 July 2023–1 June 2024). The study was conducted in accordance with the Declaration of Helsinki, and the protocol was approved by the Bioethics Committee of the Medical University in Poznań (approval no. 525/2023) on 29 March 2023. Informed consent for participation was obtained from all participants involved in the study. The study was registered at the UK’s Clinical Study Registry (ISRCTN11389213) on 22 October 2025.

### 4.1. Participants

The test group consisted of 21 participants (17 females, 4 males; mean age of 53.5 ± 7.9 years) recruited from patients diagnosed with CD according to the criteria proposed by Albanese et al. [17], presenting exclusively with caput patterns according to the Col-Cap concept [2], who were enrolled in a therapeutic programme at the department. Participation in our study was not a prerequisite for receiving botulinum toxin therapy.

All participants had been enrolled in a therapeutic programme at the department and had received BoNT-A treatment for at least 1.5 years prior to inclusion in this study, with both movement pattern and dosing remaining stable throughout this time. Laboratory testing and brain and cervical spine MRI performed at programme enrolment were used to exclude structural or systemic pathology. Stable caput patterns without tremulous, non-rhythmic, phasic, or myoclonic movements were confirmed by three experienced physicians. The distribution of caput patterns was as follows:

- 9 patients with LCap/TCap;
- 3 with LCap/TCap/ACap;
- 4 with LCap/TCap/RCap;
- 2 with TCap/ACap;
- 3 with TCap/RCap.

Exclusion criteria in the study comprised diabetes mellitus, alcohol dependence, polyneuropathy, mononeuropathy (e.g., carpal tunnel syndrome, ruled out by normal median nerve conduction), renal dysfunction, systemic inflammatory or malignant diseases, and use of psychotropic or other medications affecting nerve conduction. After confirming eligibility, all participants underwent a detailed neurological examination (assessing pain, touch, vibration sensitivity, and limb reflexes), revealing no pathological findings.

The first round of assessments was conducted 4–6 weeks after BoNT-A administration and represents the peak BoNT-A effect, whereas the second round occurred more than 14 weeks after injections, and represents the waning response phase. At this point, the therapeutic effects of BoNT-A had diminished, and patients subjectively reported an approximate 50% worsening of symptoms compared with the greatest perceived improvement following the previous injection. This delay in injection relative to the standard 12–14-week interval arose from external factors and was not intentionally introduced as part of the study design. All 21 participants completed the study, with no dropouts. No adverse events were noted in the course of the study.

### 4.2. Botulinum Toxin Treatment Protocol

The CD patients received ultrasound-guided injections of botulinum toxin type A (BoNT-A) into the muscles selected according to the Col-Cap concept [2,21]. Table 5 lists the muscles targeted for injection across the patterns examined in this studywith corresponding innervation details.

Of the 21 patients, 10 received AbobotulinumtoxinA (mean dose: 700.1 units, SD 173.2), and 11 received OnabotulinumtoxinA (mean dose: 210.9 units, SD 34.19).

### 4.3. Methods

F-wave and cutaneous silent period (CSP) testing were conducted at the Neurology Department of the Municipal Hospital in Poznań. The procedures were carried out by a technician with 10 years of experience, under the supervision of a neurophysiologist (M.D., PhD) with 25 years of experience, who also interpreted the results.

Recordings were made with a 3-channel EMG instrument Dantec Keypoint G4, type 9031A070101, manufactured by Natus Manufacturing Ltd., Galway, Ireland, year of production: 2018, using the F-wave, Motor and Sensory Nerve Conduction programmes. All recordings were performed with the following electrodes:

- Stimulating electrode (ref. nr 9013L0362), manufactured by Natus Manufacturing Ltd., Galway, Ireland;
- Recording electrode (ref. nr 9013S0242), manufactured by Ambu A/S, Ballerup, Denmark;
- Grounding electrode (ref. nr GDRGP0450326), manufactured by Spes Medica S.r.l., Genova, Italy.

The recordings were performed in a quiet room at constant temperature (22–24 °C) to maintain a minimum skin temperature of 32 °C, with participants lying on a medical couch with a relaxed, supine position of the upper limb.

The F-wave examination was followed by a 15 min break and the CSP examination.

#### 4.3.1. F-Wave and M-Wave Testing Protocol

The F-wave testing protocol was adapted from the methodologies described by Fisher et al. [32] and Puksa et al. [33]. All participants included in the study were right-handed. They underwent motor nerve conduction examination on the right upper limb and F-wave examination of the medial nerve recorded from the APB muscle. The active electrode was placed over the muscle belly, the reference electrode over the distal tendon, and the ground electrode over the hand dorsum.

During the F-wave recording, bandpass filtering was set to 20 Hz and 10 kHz, the amplifier gain to 0.5 mV/division, and the total sweep time was 5 ms/division. During the M-wave recording, bandpass filtering was set to 20 Hz and 10 kHz, the amplifier gain to 5 mV/division, and the total sweep time was 5 ms/division.

**Figure 1:**
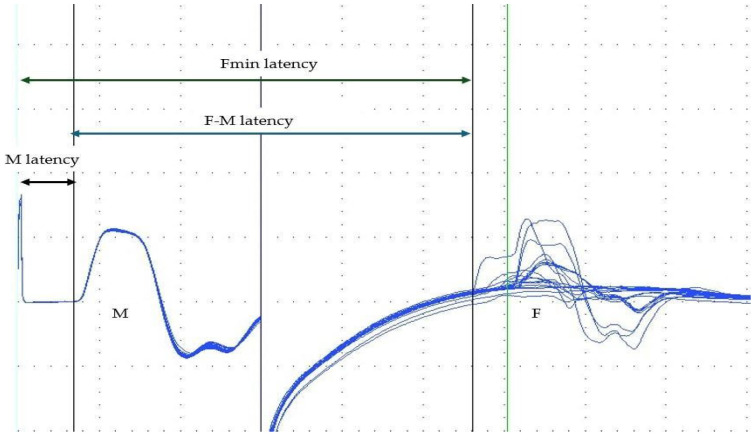
Illustration of M-wave, F-wave and M, F and F–M latencies.

The median nerve was stimulated at the wrist with a superficial bipolar electrode, using a supramaximal stimulus intensity (typically 20–30% above the level needed to evoke a maximal compound muscle action potential (CMAP)), delivered with a pulse duration of 0.2 ms. A total of 20 stimuli were delivered at a frequency of 0.5 Hz, each with a duration of 0.2 ms. The F-wave was identified as the first action potential following the M-wave, with a minimum amplitude of 100 µV. Figure 1 presents the main responses and latencies.

Table 6 provides relevant electrophysiological parameters and anatomical distances measured in the study. All distances were measured on the skin surface with a flexible tape measure supplied by the EMG manufacturer and a Breisky pelvimeter, with the upper limb in the anatomical position, in 30° humeral abduction. Measurements followed the nerve’s anatomical course: measurement on the skin surface was taken from the stimulation cathode on the wrist, through the centre of the cubital fossa to the highest point of the axillary fossa and a Breisky pelvimeter was used to measure the distance between the axilla and the Erb’s point and between the Erb’s point to C7 spinous process.

To increase the precision of F-wave conduction velocity assessment, two complementary approaches were employed. The standard measurement, CV1, was calculated using D1, defined as twice the distance measured from the cathode of the stimulating electrode at the wrist, along the forearm to the centre of the cubital fossa, then along the upper arm to the axilla, the Erb’s point and the tip of the C7 spinous process.

In addition, CV2 was calculated using D2, which equals D1 plus the distance from the stimulating cathode on the wrist to the recording electrode in the centre of the abductor pollicis brevis (APB) muscle. CV1 and CV2 were calculated by dividing D1 and D2, respectively, by the minimum F-wave latency. This dual-distance methodology was utilised to enhance the accuracy and reliability of conduction velocity estimation. Table 7 lists conduction velocities and ratios used in the study. CV1 and CV2 represent conduction velocities along the motor pathways (alpha motoneuron).

#### 4.3.2. CSP Testing Protocol

The CSP testing protocol was adapted from the methodologies described by Kofler et al. [15], Tiric-Campara et al. [34], Bölük et al. [35], and Neves et al. [36]. All participants were right-handed, and electromyographic (EMG) recordings were obtained from the right abductor pollicis brevis (APB) muscle using surface electrodes in a belly–tendon configuration. The active electrode was placed over the muscle belly, the reference electrode over the distal tendon, and the ground electrode over the hand dorsum. Skin impedance was kept below 5 kΩ.

Participants were asked to maintain a steady voluntary isometric contraction of the APB at approximately 40–50% of maximal effort.

Electrical stimulation was delivered to the digital nerve of the index finger using a bipolar stimulating electrode (Natus Manufacturing Ltd., Galway, Ireland, ref. nr 9013L0362) and a recording electrode (Ambu A/S, ref. nr 9013S0242, Ballerup, Denmark;). The stimulus intensity was set at 20 times the sensory threshold (ST), and the average sensory threshold was 1.75 ± 0.53 mA (mean ± SD), with a duration of 0.2 ms. The filter settings were identical to those in a motor conduction study (20 Hz–5 kHz), the sweep speed was 20–50 ms/division, and the sensitivity was 0.5–1 mV/division.

Each participant received 12 stimuli, with interstimulus intervals of 10–15 s to avoid habituation and temporal summation effects. The CSP was visually identified as the suppression of voluntary EMG activity following the afferent stimulus, with clear onset and offset markers. The CSP onset latency was defined as a decrease in the EMG trace below 80% of the baseline preceding the stimulus, and the duration of CSP was calculated.

For each participant, eight optimal responses were selected and averaged for analysis. Table 8 lists the electrophysiological parameters and anatomical distances used to calculate the relevant conduction velocities presented in Table 9. All distances were measured on the skin surface with a flexible tape measure supplied by the EMG manufacturer (Natus Manufacturing Ltd., Galway, Ireland) and a Breisky pelvimeter (Weldon Instruments, Sialkot, Pakistan), with the upper limb in the anatomical position, in 30° humeral abduction. Measurements followed the nerve’s anatomical course: measurement on the skin surface was taken from the stimulation cathode on the wrist, through the centre of the cubital fossa to the highest point of the axillary fossa, and a Breisky pelvimeter was used to measure the distance between the axilla and the Erb’s point and between the Erb’s point to C7 spinous process.

CV3 was calculated using D3, defined as the distance measured from the stimulating electrode on the right index finger to the middle of the wrist, along the forearm to the centre of the cubital fossa to the highest point of the axillary fossa and a Breisky pelvimeter was used to measure the distance between the axilla and the Erb’s point and between the Erb’s point to C7 spinous process.

CV4 was calculated using D4, defined as the distance measured from the recording electrode in the middle of the abductor pollicis brevis (APB) muscle to the centre of the wrist, along the forearm to the centre of the cubital fossa and along the upper arm to the highest point of the axillary fossa, and then, using a Breisky pelvimeter, from the axilla to the Erb’s point to the spinous process of C7. CV5 is the sum of D3 and D4 distances. Calculations of CV4 and CV5 conduction velocities use distances D4 and D5 and the durations of CSPd and CSPe. The CSP assessment involves a polysynaptic pathway comprising both central and peripheral components. Because it is not possible to precisely determine the relative contributions of central and peripheral conduction times in the CSP assessment, the duration of CSd and the total duration of CSPe were used.

To enhance the accuracy of assessing the conduction of spinal and peripheral pathways, a novel approach to conduction velocity measurement was implemented in this study. While previous studies typically evaluated only CSP latencies (CSPo and CSPe) and the inter-latency interval (CSPe–CSPo) without taking into account individual anatomical variation such as arm length, the current protocol explicitly addresses this limitation. Conduction velocity depends jointly on the anatomical distance traversed by neural impulses and the latency to CSP onset. Incorporating precise distance measurements enables a more reliable evaluation of the efficiency of both afferent and efferent neural pathways than latency values alone, albeit at the cost of increased procedural complexity. Building on the standard approach, CV1, pertaining to the F-wave, was calculated using the distance D1, defined as twice the length from the stimulating electrode on the wrist to process C7. To further improve measurement precision, additional conduction velocities (CV2–CV5) were derived from anatomical distances D2–D5, each accounting for distinct segments along the relevant neural pathways, as detailed in Table 6, Table 7, Table 8 and Table 9.

**Figure 2:**
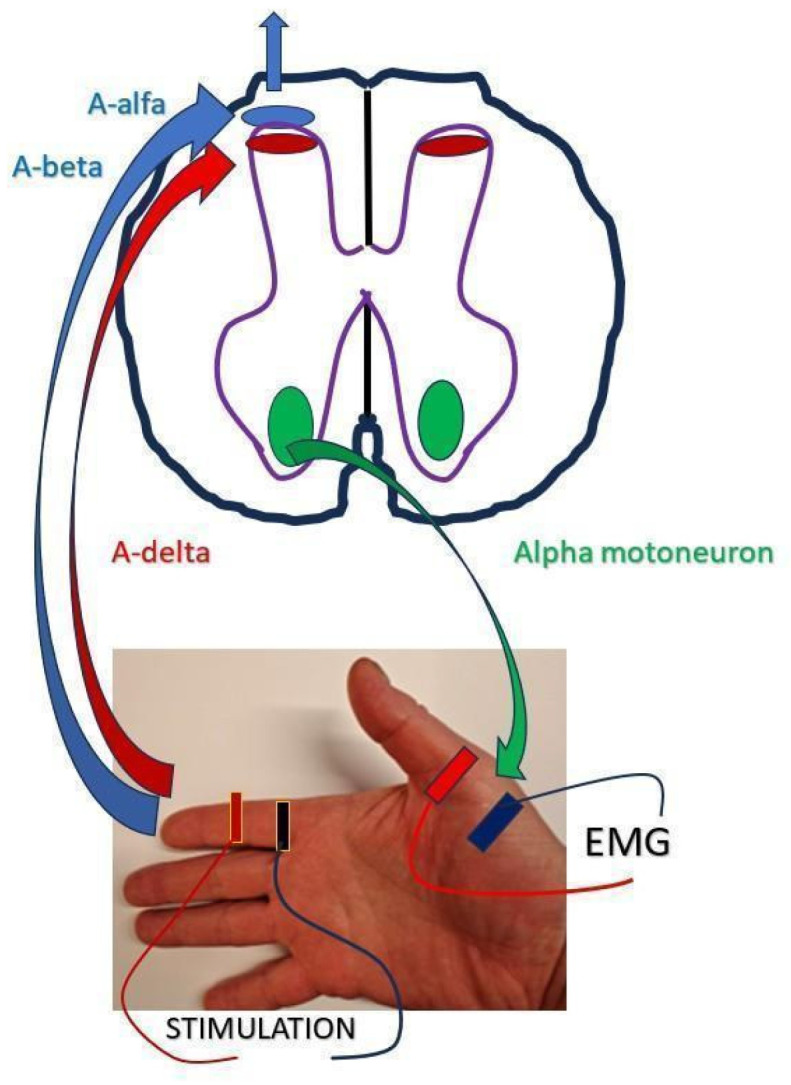
Illustration of sensory and motor pathways used for CSP testing, adapted from Kofler et al. [37].

The sensory conduction velocity (CV3) was defined as the distance between the stimulation site and C7, divided by the CSP onset latency. The motor velocity (CV4) was defined as the distance from C7 to the recording site (effector), divided by the duration of the CSPd (calculated as the difference between the CSPe and CSPo latencies). This interval reflects the processing time of spinal and supraspinal structures, as well as the excitation of the effector via alpha-motoneurons. Finally, the total conduction velocity (CV5) was defined as the combined distance from the stimulation site to C7 and from C7 to the recording site, divided by the total response time, represented by the CSPe latency. Figure 2, adapted from Kofler et al. [37], illustrates the spinal neural pathways responsible for generating the CSP. Electrical stimulation of the fingertip activates two types of afferent nerve fibres. First, the low-threshold A-alpha and A-beta fibres (thick-myelinated) are activated. When stimulation intensity increases, the high-threshold A-delta fibres (thin-myelinated) are activated. Alpha-motoneurons, along with their descending connections from the corticospinal tract, represent the efferent pathway in this circuitry.

### 4.4. Data Collection and Management

Data were collected prospectively and managed in accordance with ethical standards and Good Clinical Practice guidelines. Neurophysiological measurements were taken by two qualified and experienced technicians licenced by the Polish Society of Clinical Neurophysiology [Polskie Towarzystwo Neurofizjologii Klinicznej]). The obtained data were reviewed by a neurologist with the Polish Society of Clinical Neurophysiology credentials and manually entered into structured Excel (Excel to Microsoft Office 2019) case report forms, stored in a secure institutional database with restricted access on the hospital server. Data entry was double-checked by an external neurophysiologist, and source documentation was reviewed in case of discrepancies. All data were independently assessed by two neurophysiologists. The external neurophysiologist’s role was limited to data analysis, without any direct involvement with participants. Discrepancies were resolved by consensus.

All procedures related to data handling adhered to the institutional data protection policy and complied with the General Data Protection Regulation (GDPR) requirements. Demographic and clinical data were recorded using structured case report forms in Excel. Neurophysiological data were anonymised, coded with unique subject identifiers, and stored in a secure institutional database with restricted access on the hospital server. Regular personal data protection audits were conducted to verify completeness and accuracy. The most recent audit took place 14–18 July 2025.

### 4.5. Statistical Analysis

The normality of distribution for quantitative variables was assessed using the Shapiro–Wilk test. Variables with a normal distribution are presented as mean ± standard deviation (SD), while variables with a non-normal distribution are presented as median and interquartile range (Q1–Q3). Statistical significance was evaluated at an alpha level of 0.05. The effect size was assessed using Cohen’s d coefficient. For comparisons involving normally distributed variables, Student’s parametric *t*-test for paired samples was applied. If variables did not meet normality assumptions or were ordinal, non-parametric Wilcoxon signed-rank test for paired samples was used. Because multiple comparisons were performed, the Holm–Bonferroni correction was applied.

All analyses were performed using the MedCalc^®^ Statistical Software version 22.014 (MedCalc Software Ltd., Ostend, Belgium; https://www.medcalc.org; 2023) and PQStat Software version 1.8.6.120 (PQStat, Poznań, Poland; 2024).

## Figures and Tables

**Table 1:** F- and M-wave measurement parameters at waning and peak response phases of botulinum toxin therapy in patients with caput patterns of cervical dystonia (n = 21). (a—Student *t*-test, b—Wilcoxon, H–B—Holm–Bonferroni).

Parameter	Waning BoNT-A Mean ± SD Median (Q1–Q3)	Peak BoNT-A Mean ± SD Median (Q1–Q3)	*p*-Value	d-Cohen	p H–B
Fmin (ms)	23.70 ± 1.90 23.60 (22.05–25.15)	24.25 ± 1.88 25.00 (22.60–25.13)	0.026 ^a^	1.327	0.240
Fmax (ms)	29.22 ± 2.39 29.30 (27.38–31.20)	29.76 ± 2.67 30.00 (27.70–32.15)	0.273 ^a^	0.562	0.546
Fmean (ms)	26.48 ± 1.82 26.90 (24.93–28.00)	26.78 ± 1.95 27.35 (24.93–27.73)	0.169 ^a^	0.885	0.676
Chronodisp (ms)	5.57 ± 1.15 5.40 (4.88–6.10)	5.05 ± 0.80 4.90 (4.50–5.20)	0.073 ^b^	0.849	0.438
Fduration (ms)	12.33 ± 1.76 12.30 (11.00–13.25)	12.98 ± 2.18 13.10 (11.85–14.45)	0.235 ^a^	0.333	0.705
Fpersistence (n)	16.43 ± 2.87 17.00 (13.75–18.25)	15.52 ± 2.52 16.00 (13.75–17.25)	0.024 ^b^	1.128	0.240
Fampl (mV)	0.33 ± 0.07 0.35 (0.30–0.37)	0.27 ± 0.10 0.27 (0.21–0.35)	0.040 ^a^	0.483	0.320
Mlat (ms)	3.70 ± 0.43 3.80 (3.38–4.04)	3.66 ± 0.46 3.78 (3.34–3.96)	0.383 ^a^	0.581	0.546
Mampl (mV)	7.33 ± 1.86 7.50 (6.10–8.43)	6.91 ± 1.90 6.90 (5.53–8.15)	0.088 ^a^	0.979	0.440
Marea (ms × mV)	20.11 ± 6.03 18.10 (17.03–22.73)	18.60 ± 6.00 17.10 (14.25–23.10)	0.041 ^a^	1.280	0.320

**Table 2:** Calculated F-wave parameters at waning and peak response phases of botulinum toxin therapy in patients with caput patterns of cervical dystonia (n = 21). (a—Student *t*-test, b—Wilcoxon, H–B—Holm–Bonferroni).

Parameter	Waning BoNT-A Mean ± SD Median (Q1–Q3)	Peak BoNT-A Mean ± SD Median (Q1–Q3)	*p*-Value	d-Cohen	p H–B
F/Mampl (ratio)	4.91 ± 2.04 4.53 (3.85–5.25)	4.31 ± 2.16 3.98 (2.79–5.39)	0.099 ^b^	0.772	0.198
F/Mlat (ratio)	34.67 ± 5.04 33.49 (32.05–38.05)	35.75 ± 5.19 35.59 (32.13–40.03)	0.730 ^a^	0.465	0.730
F–Mlat (ms)	20.00 ± 1.90 20.10 (18.60–21.21)	20.59 ± 1.93 20.80 (19.22–21.83)	0.022 ^a^	1.335	0.110
CV1 (m/s)	78.83 ± 6.64 79.39 (72.29–81.31)	76.45 ± 6.70 77.65 (72.09–81.17)	0.022 ^a^	1.160	0.110
CV2 (m/s)	83.11 ± 6.87 83.62 (76.22–85.99)	80.60 ± 6.94 81.26 (76.07–85.63)	0.022 ^a^	1.160	0.110

**Table 3:** CSP measurement parameters at waning and peak response phases of botulinum toxin therapy in patients with caput patterns of cervical dystonia (n = 21). (a—Student *t*-test, H–B—Holm–Bonferroni).

Parameter	Waning BoNT-A Mean ± SD Median (Q1–Q3)	Peak BoNT-A Mean ± SD Median (Q1–Q3)	*p*-Value	d-Cohen	p H–B
CSPo (ms)	77.69 ± 12.62 72.40 (67.13–88.38)	78.15 ± 11.80 74.00 (68.90–89.25)	0.482 ^a^	0.949	0.482
CSPe (ms)	116.32 ± 10.54 115.00 (107.88–123.73)	126.84 ± 7.80 128.00 (118.98–133.25)	<0.0001 ^a^	3.953	0.00036
CSPd (ms)	38.64 ± 5.89 39.20 (33.63–42.93)	48.69 ± 7.33 48.40 (43.23–51.95)	<0.0001 ^a^	3.932	0.00036
CSPom (ms)	65.40 ± 12.93 62.00 (54.95–72.48)	68.16 ± 11.27 67.00 (60.13–73.30)	0.009 ^a^	2.703	0.018

**Table 4:** CSP-related conduction velocity parameters at peak and waning response phases of botulinum toxin therapy in patients with caput patterns of cervical dystonia (n = 21). (a—Student *t*-test, H–B—Holm–Bonferroni).

Parameter	Waning BoNT-A Mean ± SD Median (Q1–Q3)	Peak BoNT-A Mean ± SD Median (Q1–Q3)	*p*-Value	d-Cohen	p H-B
CV3—Sensory CV (m/s)	9.86 ± 1.88 10.50 (8.16–11.26)	9.77 ± 1.77 10.24 (8.49–11.18)	0.244 ^a^	2.110	0.244
CV4—Motor CV (m/s)	21.88 ± 4.00 21.64 (18.68–24.23)	17.26 ± 2.57 16.86 (15.48–19.66)	<0.0001 ^a^	3.913	0.00036
CV5—Total CV (m/s)	14.86 ± 1.99 15.04 (13.61–16.10)	13.55 ± 1.41 13.48 (12.93–14.32)	<0.0001 ^a^	4.913	0.00036

**Table 5:** Subtypes of CD with corresponding primary (P) and secondary (S) muscles recommended for injection, as classified by Jost and Tatu [22], and their innervation.

Pattern	Injection Side	Muscle	Innervation
Torticaput	Contralateral	Trapezius pars descendens (P) Sternocleidomastoideus (P) Semispinalis capitis pars med (S)	CN XI, cervical plexus [23,24,25] CN XI, cervical plexus [23,24,25] Dorsal rami of C1–C5 [26]
	Ipsilateral	Obliquus capitis inferior (P) Splenius capitis (S)	Suboccipital nerve (dorsal ramus of C1) [27] Dorsal rami of C2–C3 [26,28]
Laterocaput	Ipsilateral	Sternocleidomastoideus (P) Trapezius pars descendens (P) Splenius capitis (P) Semispinalis capitis (S) Levator scapulae (S)	CN XI, cervical plexus [23,24,25] CN XI, cervical plexus [23,24,25] Dorsal rami of C2–C3 [26,28] Dorsal rami of C1–C5 [26] Dorsal scapular nerve (C5), C3–C4 cervical nerves [29,30,31]
Anterocaput	Bilateral	Levator scapulae (P) Sternocleidomastoideus (S)	Dorsal scapular nerve (C5), C3–C4 cervical nerves [28,29,30] CN XI, cervical plexus [22,23,24]
Retrocaput	Bilateral	Obliquus capitis inferior (P) Semispinalis capitis (P) Trapezius pars descendens (P) Splenius capitis (S)	Suboccipital nerve (dorsal ramus of C1) [27] Dorsal rami of C1–C5 [25] CN XI, cervical plexus [22,23,24] Dorsal rami of C2–C3 [26,28]

**Table 6:** F-wave and M-wave: electrophysiological parameters and anatomical distances measured in the study.

Parameter Abbrev.	Parameter Description	Unit
Fmin	Minimal latency of all F-wave responses received	ms
Fmax	Maximal latency of all F-wave responses received	ms
Fmean	Calculated as Fmin + (Fmax − Fmin)/2	ms
Fchronodisp	Chronodispersion of F-waves; interval between Fmin and Fmax	ms
Fduration	Mean duration of each F-wave response	ms
Fpersistence	Number of F-wave responses observed in 20 stimulations	-
Fampl	Mean amplitude of all F-wave responses	mV
Mlat	Latency of M-wave	ms
Mampl	Maximal amplitude of M-wave	mV
Marea	Area under the curve of M-wave	ms × mV
**Distance** **Abbrev.**	**Distance description**	**Unit**
D1	Double the distance from the stimulating electrode on the wrist along the forearm and upper arm, the axilla, the Erb’s point, to the spinous process of C7.	cm
D2	D1 plus the distance from the stimulating cathode on the wrist to the recording electrode in the middle of the abductor pollicis brevis (APB) muscle.	cm

**Table 7:** F-wave and M-wave: conduction velocities and ratios calculated in the study.

Abbrev.	Description	Formula	Unit
F/Mampl	F/M amplitude ratio	Mean F-wave amplitude ÷ Maximal M-wave	-
F/Mlat	F/M latency ratio	((Fmin − Mlat − 1)/2) × Mlat	-
F–Mlat	F–M latency difference	Fmin − Mlat	ms
CV1	Conduction velocity 1 (along the motor pathways)	D1 ÷ Fmin latency	m/s
CV2	Conduction velocity 2 (along the motor pathways)	D2 ÷ Fmin latency	m/s

**Table 8:** CSP: electrophysiological parameters and anatomical distances measured in the study.

Parameter Abbrev.	Parameter Description	Unit
CSPo	Latency at the beginning of muscle activity suppression	ms
CSPe	Latency at the onset of renewed muscle activity	ms
CSPd	Duration of the silent period, Calculated as CSPe − CSPo	ms
CSPom	Onset minimal latency of voluntary suppression across eight responses	ms
**Distance** **Abbrev.**	**Distance description**	**Unit**
D3	Distance from the stimulating electrode on the right index finger to the centre of the wrist, along the forearm and the upper arm to the axilla, the Erb’s point, to the spinous process of C7	cm
D4	Distance from the recording electrode in the middle of the right abductor pollicis brevis (APB) muscle, to the centre of the wrist, along the forearm and the upper arm to the axilla, the Erb’s point, to the spinous process of C7	cm
D5	Sum of D3 and D4	cm

**Table 9:** CSP: conduction velocities calculated in the study.

Abbrev.	Description	Formula	Unit
CV3	Sensory conduction velocity	D3 ÷ CSPo	m/s
CV4	Motor (conduction) velocity (motor pathway)	D4 ÷ CSP duration	m/s
CV5	Total conduction velocity (motorsensory circuit)	D5 ÷ CSPe	m/s

## Data Availability

The database cannot be made available, as it contains patient data and is hosted on the hospital’s server, access to which is restricted under Polish law due to GDPR and limitations on external access to hospital servers. Due to the nature of the ongoing research project and institutional data governance requirements, the datasets are not publicly available at this stage. Requests for access can be directed to the first author, who will initiate the formal review process through the hosting institution. Please note that data sharing is subject to institutional and ethical approvals and may be restricted to ensure the integrity and confidentiality of the broader research programme.

## Abbreviations

The following abbreviations are used in this manuscript:

CD	cervical dystonia
CSP	cutaneous silent period
BoNT-A	botulinum toxin type A
TCap	torticaput
LCap	laterocaput
ACap	anterocaput
RCap	retrocaput
EMG	electromyography
APB	abductor pollicis brevis
